# Association of *C1QB *gene polymorphism with schizophrenia in Armenian population

**DOI:** 10.1186/1471-2350-12-126

**Published:** 2011-09-28

**Authors:** Roksana Zakharyan, Aren Khoyetsyan, Arsen Arakelyan, Anna Boyajyan, Anaida Gevorgyan, Anna Stahelova, Frantisek Mrazek, Martin Petrek

**Affiliations:** 1 Laboratory of Macromolecular Complexes, Institute of Molecular Biology, National Academy of Sciences of the Republic of Armenia, 7 Hasratyan St., 0014, Yerevan, Armenia; 2 Laboratory of Immunogenomics and Immunoproteomics, Faculty of Medicine and Dentistry, Palacky University, 6 I. P. Pavlova St., 775 20, Olomouc, Czech Republic; 3 Nork clinic attached to Psychiatric Medical Center of the Ministry of Health of the Republic of Armenia, 2a G. Hovsepyan St., 0047, Yerevan, Armenia

## Abstract

**Background:**

Schizophrenia is a complex, multifactorial psychiatric disorder. Our previous findings indicated that altered functional activity of the complement system, a major mediator of the immune response, is implicated in the pathogenesis of schizophrenia. In order to explore whether these alterations are genetically determined or not, in the present study we evaluated the possible association of complement C1Q component gene variants with susceptibility to schizophrenia in Armenian population, focusing on four frequent single nucleotide polymorphisms (SNPs) of *C1QA *and *C1QB *genes.

**Methods:**

In the present study four SNPs of the complement C1Q component genes (*C1QA*: rs292001, *C1QB *rs291982, rs631090, rs913243) were investigated in schizophrenia-affected and healthy subjects. Unrelated Caucasian individuals of Armenian nationality, 225 schizophrenic patients and the same number of age- and sex-matched healthy subjects, were genotyped. Genotyping was performed using polymerase chain reaction with sequence-specific primers (PCR-SSP) and quantitative real-time (qRT) PCR methods.

**Results:**

While there was no association between *C1QA *rs292001, *C1QB *rs913243 and rs631090 genetic variants and schizophrenia, the *C1QB *rs291982*G minor allele was significantly overrepresented in schizophrenic patients (G allele frequency 58%) when compared to healthy subjects (46%, OR = 1.64, *p*_corr _= 0.0008). Importantly, the susceptibility for schizophrenia was particularly associated with *C1QB *rs291982 GG genotype (OR = 2.5, *p*_corrected _= 9.6E-5).

**Conclusions:**

The results obtained suggest that *C1QB *gene may be considered as a relevant candidate gene for susceptibility to schizophrenia, and its rs291982*G minor allele might represent a risk factor for schizophrenia at least in Armenian population. Replication in other centers/populations is necessary to verify this conclusion.

## Background

Schizophrenia is a complex and severe psychiatric disorder manifested by a disruption in cognition and emotion along with negative (avolition, alogia, apathy, poor social functioning) and positive (hallucinations, delusions) symptoms [1,2]. According to the neurodevelopmental theory, the etiology of schizophrenia may involve pathological processes in the brain induced by environmental factors during the early stage of brain formation [reviewed in [3]]. These processes result in genetic abnormalities leading to dysfunction of specific neural network, which might contribute to the premorbid signs for the later developed schizophrenia [3]. Evidence from epidemiological and genetic studies suggest a high degree of heritability of schizophrenia and point to a number of potential candidate genes that may be perturbed early in development leading ultimately to the development of psychotic symptoms [reviewed in [3-5]]. However, the molecular etiopathomechanisms of this disorder are still unclear.

Our recent studies indicate the crucial role of the immune system in schizophrenia and provide evidence on the alterations in the major mediator of the immune response, the complement system, in pathogenesis of this disorder [6-13]. In particular, increased functional activities of the complement classical pathway and C1Q protein, the initiator of the complement classical cascade, in schizophrenia-affected subjects has been found [8,10]. These findings are of special interest accounting for a positive linkage of schizophrenia with chromosome 1p36 loci located nearby *C1QA *and *C1QB *genes (1p36.12) [14,15]. Notably, for other components of the complement association of genetic polymorphisms with schizophrenia has been demonstrated [11,16]. In addition association of genetic diversity of HLA-III encoding complement proteins with susceptibility to schizophrenia has been also shown [17]. However, no data regarding association of C1Q encoding genes (*C1QA *and *C1QB*) polymorphisms to schizophrenia have been published yet. To explore whether the alterations in C1q activity in schizophrenia are genetically determined or not, in the present study we evaluated the possible association of complement C1Q component gene variants with susceptibility to schizophrenia in Armenian population, focusing on four *C1QA *and *C1QB *intronic single nucleotide polymorphisms (SNPs); two tagged SNPs were selected by Martens et al. [2009] using HapMap project [18] and the other two were chosen according to their frequent appearance in European Caucasoid population (minor allele frequency > 10%) and their potential functional effect (gain/loss of binding site for transcription factors) [19-22]. To our knowledge, this is the first study investigating association of the *C1QA *and *C1QB *genetic variants with schizophrenia. This study might contribute to understanding the molecular pathomechanisms responsible for generation and development of schizophrenia.

## Methods

### Study population

In total, 450 unrelated Caucasian individuals of Armenian nationality living in Armenia (225 schizophrenic patients and 225 healthy subjects) were enrolled in this study. All patients (female/male: 71/154, mean age ± SD: 44.2 ± 9.8 years, age at the first-onset of illness: 26.4 ± 8.3 years, duration of illness: 19.5 ± 7.2 years, patients with/without family history of psychiatric disorders: 94/131) were diagnosed as paranoid schizophrenics (ICD-10 code: F20.0, DSM-IV-TR code: 295.30) by two independent experienced psychiatrists [1,2]. The affected subjects were recruited from the clinics of the Psychiatric Medical Center of the Ministry of Health of the Republic of Armenia (MH RA). Age- and sex-matched healthy volunteers (female/male: 71/154, mean age ± SD: 42.6 ± 9.2 years) were recruited among the staff and blood donors of the Erebouni Medical Center MH RA. Exclusion criteria for healthy subjects included psychiatric illness during lifetime, any serious neurological or endocrine disorder, any medical condition or treatment known to affect the brain, or meeting DSM-IV criteria for mental retardation as determined from the non-patient version of the Structured Clinical Interview for DSM-IV-TR Axis I Disorders [23]. Exclusion criteria for all study participants included any serious medical disorder. All subjects gave their informed consents to participate in the study, which was approved by the Ethical Committee of the Institute of Molecular Biology of the National Academy of Sciences RA.

### Genomic DNA extraction

Genomic DNA samples were isolated from fresh blood according to the standard phenol-chloroform method [24] and stored at -30°C until further use.

### Genotyping analysis

All DNA samples were genotyped for four SNPs, namely *C1QA *rs292001, *C1QB *rs913243, rs291982 and rs631090. *C1QA *rs292001, *C1QB *rs291982 and rs631090 SNPs were genotyped by polymerase chain reaction with sequence-specific primers (PCR-SSP) under the conditions described elsewhere [25]. All primers for PCR-SSP were designed using the genomic sequences in the GenBank (http://www.ncbi.nlm.nih.gov, GenBank ID:712, 713). The primer sequences for three mentioned SNPs were as follows: 1) rs292001: allele G, reverse 5'GAT GCC CGG ATG CAA ATT AC, allele A, reverse 5'GAT GCC CGG ATG CAA ATT AT, constant forward 5'AGG CTT CAG AGA CTC ACA TTC; 2) rs291982: allele T, reverse 5'ACC TTT GCC CAG ATC CAA ATT, allele G, reverse 5'ACC TTT GCC CAG ATC CAA ATG, constant forward 5'AGC CAC AAG TCC CAA TGA GA; 3) rs631090: allele T, forward 5'CAC GGA TCT CTT ACC ATT AAA T, allele C, forward 5'CAC GGA TCT CTT ACC ATT AAA C, constant reverse 5'CAT CTG TGA AAT GGG GAT GAA. The presence/absence of allele-specific amplicons was visualized by 2% agarose gel stained with ethidium bromide fluorescence in reference to a molecular weight marker. Genotypes for *C1QB *rs913243G/T SNP were determined using TaqMan SNP genotyping assay (Applied Biosystems, Assay ID C_3176751_10) according to the manufacturer's instruction. Randomly selected samples (n = 45; 10% of the total number) were amplified twice to check for confidence of genotyping, and in each case complete concordance was obtained.

### Statistical analysis

The distributions of genotypes for all investigated SNPs were checked for correspondence to the Hardy-Weinberg (H-W) equilibrium. In order to find potential relevance of the selected SNPs to schizophrenia, their allele and genotype frequencies (carriage rates) in patients and control subjects were compared. The calculations of allele and genotype frequencies were based on the observed number of genotypes. Estimated proportion of haplotypes and linkage disequilibrium (LD) between investigated loci were calculated using SNP analyzer software [26]. Maximum-likehood (ML) haplotype frequencies in patients and control subjects were estimated using an expectation-maximization (EM) algorithm [27]. The significance of differences between allele and genotype frequencies in both groups was determined using Pearson's Chi-square test. The odds ratio (OR), 95% confidence interval (CI), and Pearson's *p*-value were calculated. Statistical power of the present study was calculated according to the protocol described elsewhere [28]. *P*-values were adjusted by Bonferroni multiple correction approach [29], and those less than 0.05 were considered statistically significant.

## Results

The genotype distributions of studied SNPs in both patients and controls complied with H-W equilibrium (p > 0.05). Statistical power of the present study, indicating the difference in the *C1QB *rs291982*G allele frequency between the patients and healthy controls for the odds ratio (OR) 2.5 reached 100%, for OR = 2.0 99.9%, while for OR = 1.2 was 38.8%.

The frequencies of all studied genetic variants in the groups of schizophrenic patients and control subjects are shown in Table 1. *C1QA *rs292001 and *C1QB *rs913243 polymorphisms were almost equally represented in both groups (*p *> 0.05). By contrast, significant differences in the *C1QB *rs631090*C (22% vs 17%, OR = 1.4, 95%CI: 1.01-1.95, *p*_nominal _= 0.044) and rs291982*G (58% vs 46%, OR = 1.64, 95%CI: 1.26-2.13, *p*_nominal _= 0.0002) minor allele frequencies between schizophrenic patients and healthy subjects were found. After Bonferroni correction for the number of tested loci (n = 4), *C1QB *rs291982*G minor allele remained more frequent at significant level in schizophrenics compared to controls (*p*_corrected _= 0.0008). The overall distribution of *C1QB *rs291982 genotypes differed between the patients and controls (*p*_corrected _= 0.0004, 2 degrees of freedom, d.f.) due to the overrepresentation of rs291982 GG homozygotes among schizophrenics (36% vs 19%, *p*_nominal _= 2.4E-5, *p*_corrected _= 9.6E-5, 1 d.f., OR = 2.5, 95%Cl: 1.62-3.85). Further, the rs291982 GG genotype was associated with increased risk for schizophrenia when compared to both TG (*p*_corrected _= 0.0004) and TT (*p*_corrected _= 0.0016) genotypes. In contrast, the susceptibility to schizophrenia was almost equal in rs291982 TG and TT genotypes (*p*_nominal _= 0.82). These results suggest that the *C1QB *rs291982*G minor allele might be considered as a risk factor for schizophrenia in a homozygous status (GG genotype, recessive model), at least in Armenian population.

**Table 1:** Distribution of *C1QA/C1QB *genotypes and carriage of minor alleles in patients with schizophrenia (SCZ) and healthy subjects (Controls).

SNP ID	Genotype 1	Genotype 2	Genotype 3	Allele 1	Allele 2	Carriage
rs292001	GG	GA	AA	G	A	A

SCZ	76 (0.34)	112 (0.50)	37 (0.16)	264 (0.59)	186 (0.41)	149 (0.66)

Controls	59 (0.26)	119 (0.53)	47 (0.21)	237 (0.53)	213 (0.47)	166 (0.74)

*p*	0.170^a^				0.070^b^	0.080^c^

rs913243	GG	GT	TT	G	T	T

SCZ	68 (0.30)	123 (0.55)	34 (0.15)	259 (0.58)	191 (0.42)	157 (0.70)

Controls	64 (0.28)	124 (0.56)	37 (0.16)	254 (0.56)	196 (0.44)	161 (0.72)

*p*	0.882^a^				0.638^b^	0.679^c^

rs291982	TT	TG	GG	T	G	G

SCZ	46 (0.20)	97 (0.44)	82 (0.36)	189 (0.42)	261 (0.58)	179 (0.80)

Controls	61 (0.27)	122 (0.54)	42 (0.19)	244 (0.54)	206 (0.46)	164 (0.73)

*p*	0.0001^a^				0.0002^b^	0.097^c^

rs631090	TT	TC	CC	T	C	C

SCZ	135 (0.60)	79 (0.35)	11 (0.05)	349 (0.78)	101 (0.22)	90 (0.40)

Controls	153 (0.68)	67 (0.30)	5 (0.02)	373 (0.83)	77 (0.17)	72 (0.32)

*p*	0.113^a^				0.044^b^	0.077^c^

The data are presented as absolute numbers with proportions (%) in parentheses.

^a ^nominal *p*-values for comparison of overall distribution of *C1QA/C1QB *genotypes between SCZ and Controls (2 degrees of freedom);

^b ^nominal *p*-values for comparison of Allele 2 proportion between SCZ and Controls;

^c ^nominal *p*-values for comparison of Allele 2 carriage between SCZ and Controls.

Haplotype analysis was performed to confirm the observed association and to estimate the ML *C1QA*/*C1QB *(rs292001/rs913243/rs291982/rs631090) haplotype frequencies. In total, 16 haplotypes in each group of study subjects were estimated. Interestingly, haplotype comparison between patients and healthy controls revealed five haplotypes with nominally significant differences between schizophrenic patients and controls, namely, A-T-T-T (haplotype frequencies in patients vs controls: 13% vs 19%, OR = 0.57, 95%CI: 0.40-0.81, *p_nominal _*= 0.02, *p_corrected _*= 0.32), A-G-T-T (4% vs 11%, OR = 0.41, 95%CI: 0.23-0.74, *p_nominal _*= 0.002, *p_corrected _*= 0.03), A-G-G-T (8% vs 4%, OR = 2.02, 95%CI: 1.13-3.63, *p_nominal _*= 0.015, *p_corrected _*= 0.24), G-G-G-C (8% versus 2%, OR = 1.99, 95%CI: 1.03-3.84, *p_nominal _*= 0.049, *p_corrected _*= 0.78), and A-T-G-C (1% vs 0.6%, OR = 4.43, 95%CI: 1.25-15.66, *p_nominal _*= 0.009, *p_corrected _*= 0.14), only one of which (A-G-T-T) remained significant after the correction for the number of haplotypes. The difference in A-G-T-T haplotype between patients and controls suggested that the *C1QB *rs291982 major T allele is less frequent in patients with schizophrenia than in controls. Importantly, these data are in complete concordance with major allele frequencies in both groups. The haplotype analysis, therefore, confirmed detected association of the *C1QB *rs291982 variant with schizophrenia.

**Figure 1:**
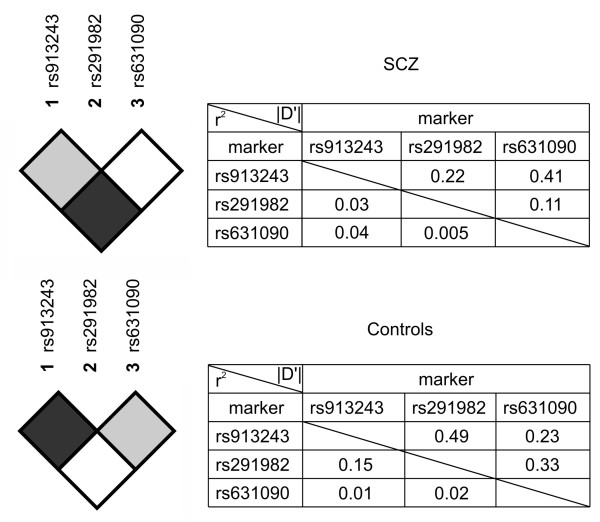
**LD blocks of *C1QB *gene rs913243/rs291982/rs631090 SNPs and values of absolute D' as well as r^2 ^for patients with schizophrenia (SCZ) and healthy subjects (Controls)**.

In both patients and controls pair-wise linkage disequilibrium (LD) between the investigated *C1QB *rs913243, rs291982, and rs631090 SNPs was determined. LD blocks of *C1QB *gene SNPs and values of absolute D' as well as r^2 ^for both groups are presented in Figure 1.

## Discussion

In this study the possible association of several polymorphisms of the complement C1Q component gene with susceptibility to schizophrenia was evaluated. No association of *C1QA *rs292001, *C1QB *rs913243 and rs631090 variants with disease was observed, whereas positive association of *C1QB *rs291982*G variant and namely GG genotype with schizophrenia was found. The results suggest that the *C1QB *rs291982*G minor allele might be in a homozygous status a risk factor for schizophrenia at least in Armenian population. Therefore, *C1QB *gene may be considered as a relevant candidate gene for susceptibility to schizophrenia.

The functionality of *C1QA *rs292001 and *C1QB *rs631090 has been previously shown in lupus erythematosus [18]. Nevertheless, our study revealed no association of these two functional variants with schizophrenia. At present, there are no published data on functionality of the *C1QB *rs291982 and rs913243 polymorphisms. In order to explore possible functional role of these genetic variants, we use the web server ALGGEN, a convenient tool for identification of the functional effects of SNPs [30]. Analysis showed that both *C1QB *rs291982 and rs913243 SNPs were found to be intronic enhancers. Thus, the *C1QB *rs291982 minor G allele contains binding sites for transcription factors forkhead box P3 (FOXP3, also known as hepocyte nuclear factor 3 (HNF3), forkhead homologue 2 or HFH-2), progesterone receptor isoform A (PRA) and B (PRB), absent in the T major allele. Further, the rs913243 G/T polymorphism of the *C1QB *gene leads to loss of binding sites of glucocorticoid receptor-alpha and retinoid × receptor-alpha transcription factors. Whereas these types of functional effects have low phenotypic risk [31], the results of our analysis might indicate the functionality of the *C1QB *rs291982 and rs913243 SNPs as enhancers, transcription-stimulating DNA regulatory elements [32]. Importantly, these transcription factors might have an indirect impact on the immune system [19-22], therefore, might contribute to the etiology of schizophrenia taken into account immune system alterations in this disease [6-13].

The association between the *C1QB *rs291982*G genetic variant and schizophrenia obtained in our study may suggest the etiological significance of *C1QB *gene in schizophrenia in Armenian population. Furthermore, this finding may reflect implication of neurodegenerative component in schizophrenia [3]. Thus, Grewal et al (1999) demonstrated overexpression of *C1QB *in the areas undergoing neurodegeneration [33]. In addition, recent studies showing alterations in expression of *C1QB *gene in hippocampus and cerebral cortex in the animal models of Alzheimer's disease [33,34], having some clinical features shared with schizophrenia such as neuron loss and cognitive impairment [1].

There may be another possible explanation of the association observed in our study in the light of the vascular-inflammatory theory of schizophrenia [35]. This theory is based on observations that inflammatory vascular disease of the brain leads to psychosis and exhibits a fluctuating course as seen in schizophrenia, and that disturbances of central nervous system blood flow have repeatedly been observed in people with schizophrenia [35]. Interestingly, it has been demonstrated that the human cerebrovascular smooth muscle cells (HCSMC) isolated from cortical vessels derived from postmortem brains can express mRNAs for complement *C1QB *gene. As HCSMC are closely associated with amyloid beta deposits in vessels in the brain, their production of complement proteins could amplify the proinflammatory effects of amyloid in the perivascular environment, further compromising brain vascular integrity [36].

Also, the observed differences in the *C1QB *gene LD blocks between the patients with schizophrenia and control subjects may suggest that *C1QB *rs291982*G allele is a marker of a haplotype carrying "causative" variant located nearby on the first chromosome. In this context it is quite important to note that chromosomal region 1p36 carrying *C1QB *gene has already been associated with schizophrenia in two genome-wide linkage scans [14,15].

## Conclusions

The results obtained suggest that *C1QB *gene may be considered as a relevant candidate gene for susceptibility to schizophrenia, and its rs291982*G minor allele might represent a risk factor for schizophrenia at least in Armenian population.

## Limitations of our study

The first limitation of our study is related to the relatively small sample size of both study groups (225 patients with schizophrenia and 225 healthy subjects), which, however, accounting for total population of Armenia (about 2,800,000) and the incidence of schizophrenia (1%) is nearly optimal for this population. In addition, the statistical power of this study, indicating the difference in the *C1QB *rs291982*G allele frequency between the patients and healthy controls for the OR = 2.5 reached 100%, for OR = 2.0 99.9%, while for OR = 1.2 was 38.8%. Secondly, we did not reveal how the *C1QB *rs291982 SNP, significantly associated with schizophrenia in our study, alters the C1QB function. Replication of these data in other centres/populations is required to confirm our results, and also, in the future investigations of functional effects of this genetic variant would be desirable.

## Competing interests

The authors declare that they have no competing interests.

## Authors' contributions

RZ performed extraction of DNA and was responsible for genotyping analyses and drafting of the manuscript. AK selected polymorphisms of studied genes, AA performed haplotype analysis. AB generated the main idea of the study. AG was responsible for selection and diagnosis of schizophrenia patients, and the organization of interviews with diseased and healthy subjects. AS designed the primers for selected polymorphisms. MP created study's laboratory design. Statistical analysis was performed by FM, who together with RZ and MP interpreted the data. AB, FM and MP finalized the manuscript. All authors read and approved the manuscript.

## Pre-publication history

The pre-publication history for this paper can be accessed here:

http://www.biomedcentral.com/1471-2350/12/126/prepub
